# PSFHSP-Net: an efficient lightweight network for identifying pubic symphysis-fetal head standard plane from intrapartum ultrasound images

**DOI:** 10.1007/s11517-024-03111-1

**Published:** 2024-05-09

**Authors:** Ruiyu Qiu, Mengqiang Zhou, Jieyun Bai, Yaosheng Lu, Huijin Wang

**Affiliations:** 1 Department of Electronic Engineering, College of Information Science and Technology, Jinan University, Guangzhou, 510632 China; 2 Guangdong Provincial Key Laboratory of Traditional Chinese Medicine Information Technology, Jinan University, Guangzhou, 510632 China; 3 Department of Computer Science, College of Information Science and Technology, Jinan University, Guangzhou, 510632 China

**Keywords:** Intrapartum ultrasound, Lightweight network, Ultrasound standard plane detection, Classification, Angle of progression

## Abstract

The accurate selection of the ultrasound plane for the fetal head and pubic symphysis is critical for precisely measuring the angle of progression. The traditional method depends heavily on sonographers manually selecting the imaging plane. This process is not only time-intensive and laborious but also prone to variability based on the clinicians’ expertise. Consequently, there is a significant need for an automated method driven by artificial intelligence. To enhance the efficiency and accuracy of identifying the pubic symphysis-fetal head standard plane (PSFHSP), we proposed a streamlined neural network, PSFHSP-Net, based on a modified version of ResNet-18. This network comprises a single convolutional layer and three residual blocks designed to mitigate noise interference and bolster feature extraction capabilities. The model’s adaptability was further refined by expanding the shared feature layer into task-specific layers. We assessed its performance against both traditional heavyweight and other lightweight models by evaluating metrics such as *F*1-score, accuracy (ACC), recall, precision, area under the ROC curve (AUC), model parameter count, and frames per second (FPS). The PSFHSP-Net recorded an ACC of 0.8995, an *F*1-score of 0.9075, a recall of 0.9191, and a precision of 0.9022. This model surpassed other heavyweight and lightweight models in these metrics. Notably, it featured the smallest model size (1.48 MB) and the highest processing speed (65.7909 FPS), meeting the real-time processing criterion of over 24 images per second. While the AUC of our model was 0.930, slightly lower than that of ResNet34 (0.935), it showed a marked improvement over ResNet-18 in testing, with increases in ACC and *F*1-score of 0.0435 and 0.0306, respectively. However, precision saw a slight decrease from 0.9184 to 0.9022, a reduction of 0.0162. Despite these trade-offs, the compression of the model significantly reduced its size from 42.64 to 1.48 MB and increased its inference speed by 4.4753 to 65.7909 FPS. The results confirm that the PSFHSP-Net is capable of swiftly and effectively identifying the PSFHSP, thereby facilitating accurate measurements of the angle of progression. This development represents a significant advancement in automating fetal imaging analysis, promising enhanced consistency and reduced operator dependency in clinical settings.

**Graphical abstract:**

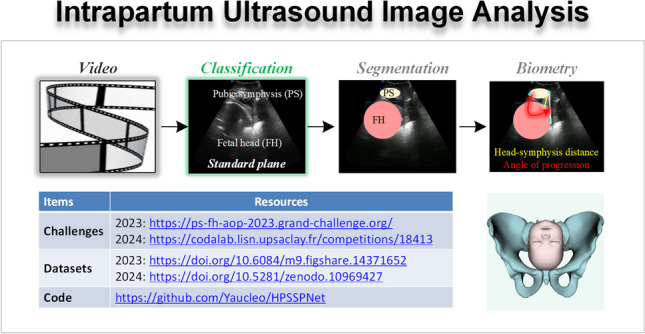

## Introduction

Ultrasound (US) imaging is extensively utilized in obstetric assessments due to its non-invasive nature, absence of radiation, affordability, and capability for real-time imaging [1]. US imaging offers a clear advantage over traditional vaginal digital examinations by providing immediate, detailed visual insights into labor progression, including cervical dilation, fetal orientation, descent of the fetal head, and the rate of labor progression [2]. These details are critical for physicians to make informed decisions about delivery management, potentially reducing neonatal mortality rates. The process of intrapartum US imaging is typically segmented into five steps: scanning, identification of the standard plane, observation of structures, measurement of parameters, and diagnosis [3]. Among these, identifying the standard plane is vital, as it must include all critical structures necessary for accurate parameter measurements, directly impacting labor evaluation [4].

For instance, the angle of progression (AoP) is an essential metric in assessing the cephalopelvic fit during labor [5]. Accurate AoP measurement requires clear visualization of the longitudinal sagittal plane of the pubic symphysis (PS) and the fetal head in US images [3]. Real-time AoP monitoring is instrumental in predicting the mode of delivery, guiding clinical interventions, and minimizing risks to both mother and infant [2]. Consequently, accurate identification of the pubic symphysis-fetal head standard plane (PSFHSP), defined as the intrapartum US image that distinctly portrays the fetal head and PS structures, is crucial for precise AoP measurements.

**Fig. 1:**
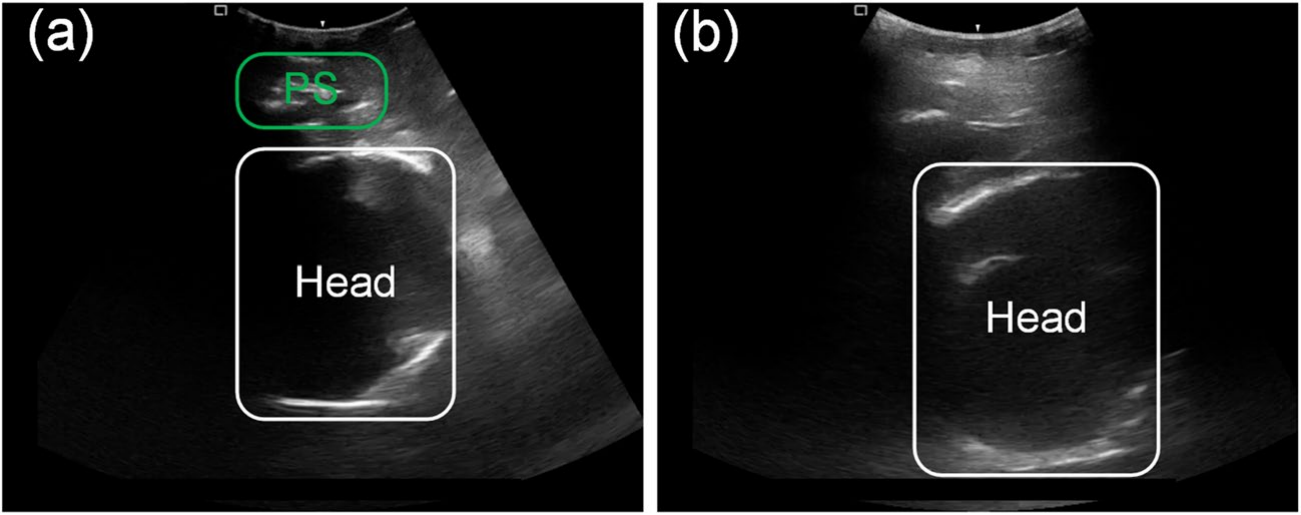
Examples of PSFHSP **(a)** and non- PSFHSP **(b)**. The structure of the PS is not clear in the non-standard plane, and its location cannot be determined; PSFHSP, pubic symphysis-fetal head standard plane; PS, pubic symphysis

However, pinpointing the PSFHSP can be a complex and laborious task for even the most skilled sonographers, heavily reliant on their expertise and practical experience [6]. The challenge is compounded by the subtle differences between PSFHSP (Fig. 1a) and non-PSFHSP images (Fig. 1b), where the high similarity between such images often complicates the determination process [7, 8]. Therefore, developing an effective, automatic, and comprehensive method to assist sonographers in swiftly and accurately identifying PSFHSP is of great importance.

## Related works

Recent discussions on the automatic identification of standard planes in ultrasound (US) imaging have highlighted various methodologies and their limitations. Yeh et al. utilized a gray-level co-occurrence matrix along with wavelet decomposition for feature extraction, followed by a support vector machine for feature classification [9]. Lei et al. examined the automatic recognition of fetal facial standard planes using Fisher vectors combined with a support vector machine on a small dataset [10]. Furthermore, Ni et al. introduced a novel method to automatically locate the fetal abdominal standard plane in sequential two-dimensional US images, initially using an AdaBoost classifier to identify the abdominal region, thereby narrowing the search area for crucial anatomical structures, followed by employing specific selection strategies for potential detection sites [11]. The primary constraints of these conventional methods are threefold: (1) feature extraction often relies heavily on manual expertise and scientific insight, (2) the scope and variety of extracted features are restricted due to manual selection processes, and (3) the necessity to reselect and re-extract features whenever the dataset is altered.

However, advancements in computational power and the accumulation of medical imaging data have facilitated the integration of deep learning technologies into the recognition of standard US images. These approaches excel at learning features from extensive datasets. For instance, Qu et al. developed a differential convolutional neural network (differential-CNN) to discern between fetal brain standard planes and non-standard planes [12]. Pu et al. crafted an automatic fetal ultrasound standard plane recognition (FUSPR) model suited for the Industrial Internet of Things (IIoT) environment, incorporating CNN and RNN fusion techniques to enhance spatial and motion analysis within video streams [13]. Zhang et al. leveraged state-of-the-art GANs for fetal brain image generation, enhancing data augmentation techniques for classifiers [14]. Moshfegh et al. presented a novel method employing a CNN to automatically detect and locate 13 standard fetal views through bounding boxes [15].

Despite these advancements, several challenges persist, primarily the small size of datasets used for training—often fewer than 1000 images, and in some instances, less than 300. Additionally, the computational costs associated with training and deploying deep learning models are seldom reported, which restricts the practical implementation of these algorithms on edge computing devices [16].

In response, we propose PSFHSP-Net, a streamlined, efficient, and end-to-end network for PSFHSP identification. By compressing ResNet18, we managed to decrease the model size without sacrificing performance, enabling deployment on edge devices for real-time applications. Moreover, we utilized the intrapartum US dataset from the Intelligent Fetal Monitoring Laboratory at Jinan University (JNU-IFM), which contains 6224 2D ultrasound images, to enhance the robustness of our model. Our method outperformed existing techniques, demonstrating superior accuracy in PSFHSP recognition.

## Materials and methods

Using the JNU-IFM dataset [7] (Section 3.2), we trained our proposed network, as detailed in Section 3.3, which is based on a compressed ResNet-18 architecture for the identification of PSFHSP.

### General

The study received ethical approval from the Medical Ethics Committee of NanFang Hospital of Southern Medical University (NFCE-2019-024). Patient data were collected under a standard-of-care clinical protocol approved by the Institutional Review Board, which waived the requirement for informed consent from all participants. This research adhered to the principles outlined in the Declaration of Helsinki, ensuring the ethical standards and integrity of the study.

### Dataset

**Fig. 2:**
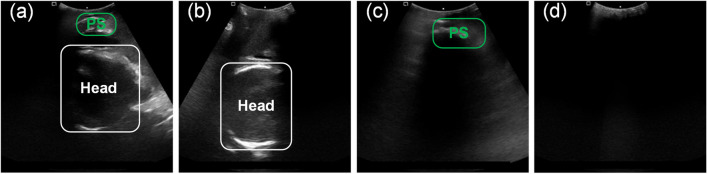
Four types of images in the JNU-IFM dataset. **a** PSFHSP: Head & PS; **b** Non-PSFHSP: Only Head; **c** Non-PSFHSP: Only PS; **d** Non-PSFHSP: None. PSFHSP, pubic symphysis-fetal head standard plane; PS, pubic symphysis

The JNU-IFM dataset, employed in this experiment, comprises intrapartum ultrasound (US) images along with corresponding segmentation labels of the pubic symphysis (PS) and fetal head. It categorizes images into four distinct groups: images featuring both PS and fetal head (PS & Head), images without PS and fetal head (None), images with only PS (Only PS), and images with only the fetal head (Only Head) as illustrated in Fig. 2a–d. For image acquisition, the ultrasound transducer was prepared by enveloping it with a surgical latex glove filled with coupling gel. This prepared transducer was then positioned between the labia below the PS to capture a sagittal plane. Fine adjustments, such as lateral movements of the probe, were made until images clearly displaying maternal pelvic and fetal landmarks without shadows from the pubic rami were obtained.

A total of 78 videos from 51 pregnant women were collected at the NanFang Hospital of Southern Medical University between 2019 and early 2020. From these videos, 6224 images were extracted at 10 frames per video and labeled using the Pair software. To facilitate balanced training and evaluation, the images were divided into training, validation, and test groups in an approximate ratio of 4:1:1, using a strategy of five-fold cross-validation to ensure each fold was as equal as possible in data amount, despite variances in individual patient data contributions.

The number distribution of images in the JNU-IFM dataset is detailed in Table 1. Given GPU memory constraints, all data were resized to 224 × 224 pixels before training. Additionally, to leverage pre-trained models effectively, which typically require three-channel input, the grayscale images were converted to RGB format for the experiments.

**Table 1:** The numbers of PSFHSP and Non-PSFHSP in training, validation, and testing datasets

	Train + validate	Test	Train + validate + test
PSFHSP	3170	413	3583
Non-PSFHSP	2068	573	2641
Sum	5238	986	6224

### PSFHSP-Net

**Fig. 3:**
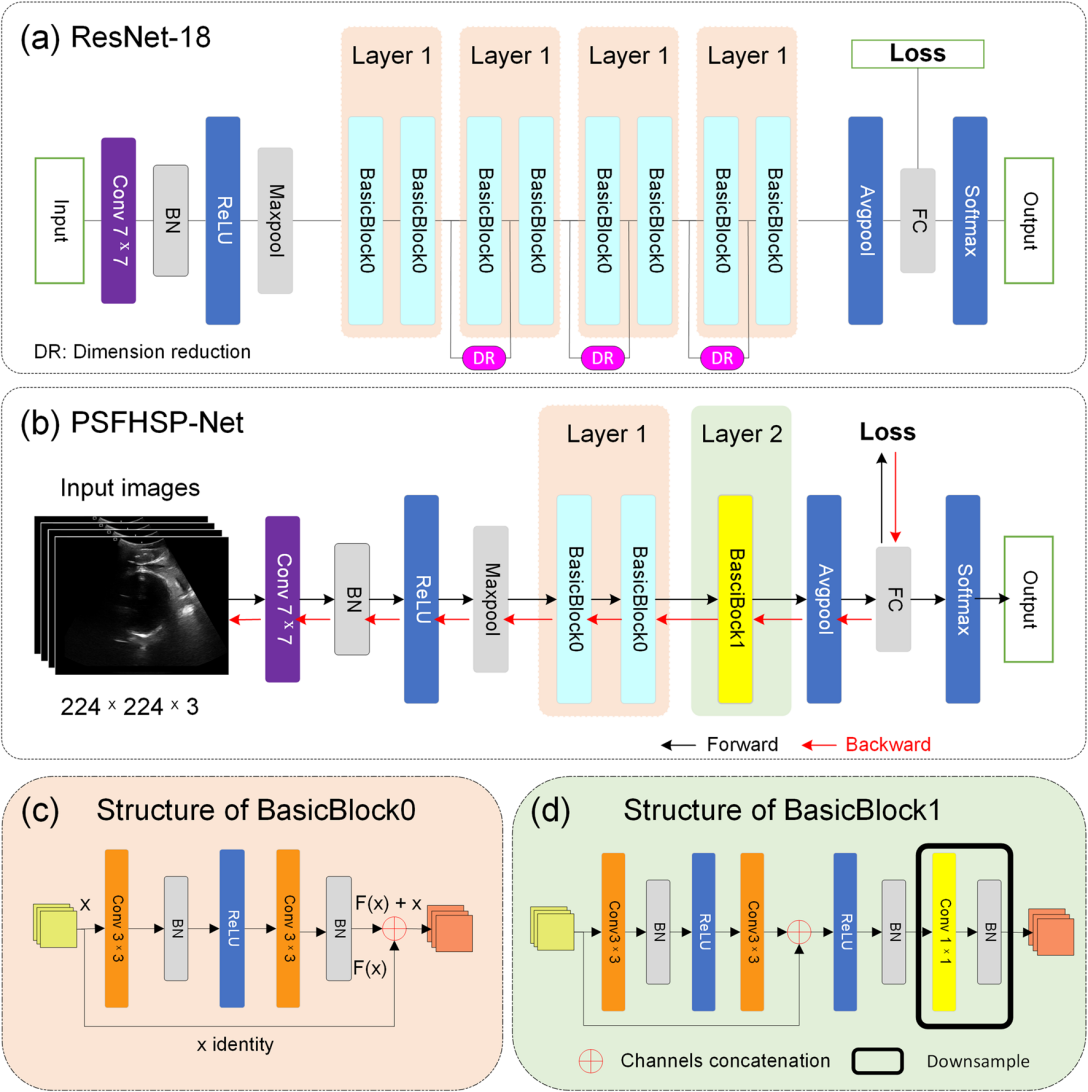
Schematic of the PSFHSP-Net architecture based on ResNet-18. **a** Structure of ResNet-18. **b** Structure of PSFHSP-Net. **c** Structure of BasicBlock0. **d** Structure of BasicBlock1. BN, batch-normalization; Conv, convolution; ReLU, Rectified Linear Unit

Addressing the challenges presented by image quality in ultrasound (US) imaging, we have developed a novel convolutional neural network (CNN) that builds on the foundational architecture of ResNet-18 (Fig. 3a). Our model incorporates a simplified structure aimed at reducing noise interference and improving analytical accuracy. Unlike the traditional ResNet-18, which might lose crucial data through repeated convolutions and downsampling—such as the lower edge of the fetal head—our design makes strategic alterations to preserve important features. Our model, designated as PSFHSP-Net, includes a 7 × 7 convolutional layer (Conv 7 × 7), two basic residual building blocks (BasicBlock0), and one modified residual building block (BasicBlock1) with only one downsampling module. A significant adjustment in our approach is the omission of three-dimension reduction (DR) modules between the BasicBlock0s, which is depicted in the schematic framework shown in Fig. 3b. The initial step involves cropping the original US image to 224 × 224 × 3 and feeding it into the network where the residual blocks extract image features before passing them to a fully connected layer. During the training phase, we compute the loss between the output of the fully connected layer and the image category label. The network parameters are then fine-tuned through gradient backpropagation [17]. In the testing phase, the fully connected layer sends the output to a classifier that determines whether an image qualifies as PSFHSP, and this result is outputted accordingly.

The early stages of the network involve a 7 × 7 convolutional layer used to downsample the input image, followed by batch normalization (BN) to stabilize the data distribution [18]. Non-linear activation is performed using the ReLU function to prevent vanishing gradients and reduce overfitting. Subsequently, the MaxPool operation expands the field of perception, removes redundant information, and highlights critical structures such as the fetal head and pubic symphysis, aiding in feature extraction.

The core feature extraction comprises two layers: Layer1 contains two BasicBlock0 units (Fig. 3c), and Layer2 consists of one BasicBlock1 (Fig. 3d). The design utilizes the principle of residual blocks [19], which involves direct mappings between different network layers to transfer lower-layer features to higher layers, effectively addressing the issue of deep network degradation. The residual block is to connect different layers of the network by direct mapping and transfer the features of the lower layer to the higher layer to solve the problem of deep network degradation. In terms of computational principles, the desired underlying mapping is represented as *H*(*x*), and the stacked nonlinear layers are represented as another mapping of *F*(*x*) = *H*(*x*) − *x*; then, the original mapping is recast as *F*(*x*) + *x*. If the learning effect of the new layer is very poor, then, we can “skip” this part directly by a residual edge. In this way, no matter how many layers there are in the network, we keep the ones that work well and skip the ones that do not, ensuring that they are at least as good as the original ones. This structure is called a short connection, and these short connections can skip two or more layers and perform the mapping directly. The residual unit equation is defined as:

1 $${X}_{l+1}=f\left({X}_l+F\left({X}_l,{k}_l\right)\right)$$

where *X*_*l*_ and *X*_*l* + 1_ represent the input and output of the *l-*th residual unit, respectively, *f* represents the activation function, *F* represents the residual function, and *k*_*l*_ represents the convolution kernel in the *l-*th residual unit.

BasicBlock0 features two 3 × 3 convolutional layers connected by a ReLU function and a BN layer, with a residual connection after the second BN layer. Unlike BasicBlock0, BasicBlock1 incorporates a 1 × 1 convolutional layer (Conv 1 × 1) in the downsampling module after reactivation, which serves as a projection layer to manage the proliferation of parameters in deeper networks and focus on retaining relevant information [20].

Post-feature extraction, an AvgPool operation, consolidates global information from the last convolutional layer into the feature map, enhancing model robustness and category correspondence. The fully connected layer then converts this two-dimensional feature map into a one-dimensional vector to facilitate end-to-end learning. Finally, a softmax classifier [21] interprets this vector to classify the input US image as PSFHSP or non-PSFHSP, ensuring precise identification of standard planes.

### Experimental settings

Our experiments were conducted using the PyTorch framework on an NVIDIA GTX 3090 GPU. We employed the Adam optimizer, with an initial learning rate set at 0.001. The model training was configured for 200 epochs with a batch size of 64. To enhance the model’s generalization and feature representation capabilities, we initialized our network with pre-trained ImageNet weights and employed a five-fold cross-validation strategy. To prevent overfitting, an early stopping mechanism was utilized, actively monitoring the loss on the validation set to adjust parameters and halt training when loss was minimized, thereby optimizing the model’s generalization.

Given the unique patient characteristics, the training-to-validation set ratio was set at 1:4. Every five epochs constituted a cycle of the cross-validation experiments. During each cycle, the average accuracy, *F*1-score, and loss were calculated, and weights achieving the highest *F*1-score were preserved. The best *F*1-score obtained on the validation set was selected as the final model representation.

During the training phase, data augmentation techniques were strategically applied. These included random horizontal and vertical flips, fixed-size cropping, and rotations. Due to the class imbalance in the PSFHSP US images and the essential nature of the depicted structures, care was taken to ensure that cropping did not omit critical features. Additionally, considering the inherent speckle noise and artifacts in US images, these augmentations were chosen to avoid exacerbating the difficulty of feature learning. The original grayscale ultrasound images were converted to RGB format for two reasons: to utilize pre-trained model weights more effectively and as a form of data enhancement, allowing the model to discern more image details through color gamut transformation.

In the testing stage, the performance of the PSFHSP-Net was assessed using a dataset of 986 images. To benchmark our model’s efficacy, we compared it against a range of established classification models under identical experimental conditions. These models included non-residual networks such as AlexNet and ZFNet; residual networks like ResNet-18, ResNet-34, ResNet-50, ResNet-101, and ResNet-152; and lightweight networks such as MobileNet [22], SqueezeNet [23], and ShuffleNet [24]. Comparative evaluation focused on metrics such as accuracy (ACC), precision, recall, *F*1-score, the number of model parameters, and inference time, enabling a comprehensive analysis of model performance across different architectures.

### Evaluation metrics

To evaluate the classification performance of the PSFHSP-Net and compare it with other models, several key metrics were utilized, including *F*1-score, accuracy (ACC), recall, precision, and the area under the receiver operating characteristic curve (AUC), as well as the number of model parameters and inference time. Here is a detailed explanation of each metric and the formulas used to calculate them:

2 $$\textrm{ACC}=\frac{\textrm{TP}+\textrm{TN}}{\textrm{TP}+\textrm{FP}+\textrm{TN}+\textrm{FN}}$$

3 $$\textrm{Precision}=\frac{\textrm{TP}}{\textrm{TP}+\textrm{FP}}$$

4 $$\textrm{Recall}=\frac{\textrm{TP}}{\textrm{TP}+\textrm{FN}}$$

5 $$F1=\frac{2\ast \textrm{Precision}\ast \textrm{Recall}}{\textrm{Precision}+\textrm{Recall}}$$

where TP, FN, FP, and TN represent the number of actually positive samples predicted to be positive samples, the number of positive samples predicted to be negative, the number of samples that are negative but predicted to be positive, and the number of actually negative samples predicted to be negative samples, respectively. TP + FN is the number of all actual positive samples, while TP + FP is the number of all predicted positive samples.

### Statistical analysis

Wilcoxon rank-sum tests were employed to compare the proposed PSFHSP-Net and other models. A *p* value less than 0.05 is considered significant. Statistical analysis was performed using SPSS 22.0 (SPSS Inc., Chicago, IL, USA).

## Results

In this section, we utilized a test dataset comprising 986 images to evaluate and analyze the classification performance of various pre-trained networks. The metrics used for assessment included accuracy (ACC), precision, recall, *F*1-score, the number of parameters, and inference time. The comparative performance of the different models is detailed in Table 2, where our PSFHSP-Net was benchmarked against non-residual networks such as AlexNet and its variant ZFNet, residual networks including ResNet-18, ResNet-34, ResNet-50, ResNet-101, and ResNet-152, and lightweight networks like MobileNetV2, MobileNetV3, SqueezeNet, and ShuffleNetV2.

**Table 2:** The performance of different models on the test dataset. The best results are in bold

Model	ACC	*F*1-score	Recall	Precision	Parameters (MB)	Frames per second (FPS)
AlexNet	0.7002 ± 0.0211	0.7464 ± 0.0489	0.6394 ± 0.1578	0.7711 ± 0.0632	217.48	56.6334 ± 2.5036
ZFNet	0.7252 ± 0.0963	0.7530 ± 0.0507	0.7231 ± 0.08620	0.7915 ± 0.1164	55.60	61.8182 ± 4.9260
ResNet-18	0.8560 ± 0.0418	0.8769 ± 0.0480	0.8586 ± 0.0643	**0.9184 ± 0.2120**	42.64	61.3156 ± 3.1925
ResNet-34	0.8412 ± 0.0724	0.8126 ± 0.1035	0.0.7211 ± 0.1433	0.8616 ± 0.0159	81.20	60.8507 ± 2.6219
ResNet-50	0.7626 ± 0.0288	0.7850 ± 0.0315	0.7219 ± 0.0518	0.8522 ± 0.0089	89.69	59.6525 ± 1.7396
ResNet101	0.7571 ± 0.0561	0.7687 ± 0.0734	0.6984 ± 0.1219	0.8721 ± 0.0227	162.14	58.3739 ± 7.4722
ResNet152	0.7657 ± 0.0648	0.7834 ± 0.0751	0.7394 ± 0.1437	0.8515 ± 0.0213	221.82	56.1523 ± 7.2858
MobileNetV2	0.7048 ± 0.0203	0.7271 ± 0.0151	0.6578 ± 0.0172	0.8141 ± 0.0328	8.49	63.8746 ± 5.4228
MobileNetV3	0.7526 ± 0.0100	0.7704 ± 0.0045	0.7212 ± 0.0016	0.8824 ± 0.0064	5.80	64.3324 ± 2.1226
SqueezeNet	0.7755 ± 0.0371	0.7924 ± 0.0504	0.7386 ± 0.1213	0.8737 ± 0.0367	2.81	64.3553 ± 3.1159
ShuffleNetV2	0.8388 ± 0.0373	0.8773 ± 0.0444	**0.9812 ± 0.0743**	0.8532 ± 0.0197	4.79	65.1489 ± 4.3679
PSFHSP-Net	**0.8995 ± 0.0226**	**0.9075 ± 0.0282**	0.9181 ± 0.0880	0.9022 ± 0.0857	**1.51**	**65.7909 ± 1.9000**

As shown in Table 2, the performance of our proposed model on the test dataset demonstrated a mean ACC of 0.8995 and an *F*1-score of 0.9075, significantly surpassing the other models. The recall of PSFHSP-Net, at 0.9181, was lower than that of ShuffleNetV2 (0.9812), and its precision, at 0.9022, was slightly lower than ResNet-18’s 0.9184; however, both metrics were consistently above 0.90. Notably, the parameter size of PSFHSP-Net (1.51 MB) is smaller than that of the ultra-lightweight SqueezeNet (2.81 MB), commonly used on mobile devices, indicating significant advantages in terms of hardware resource consumption. Additionally, the frame rate of PSFHSP-Net (65.7909 frames per second) surpasses other models, thereby fulfilling the real-time requirement of more than 24 images per second.

**Fig. 4:**
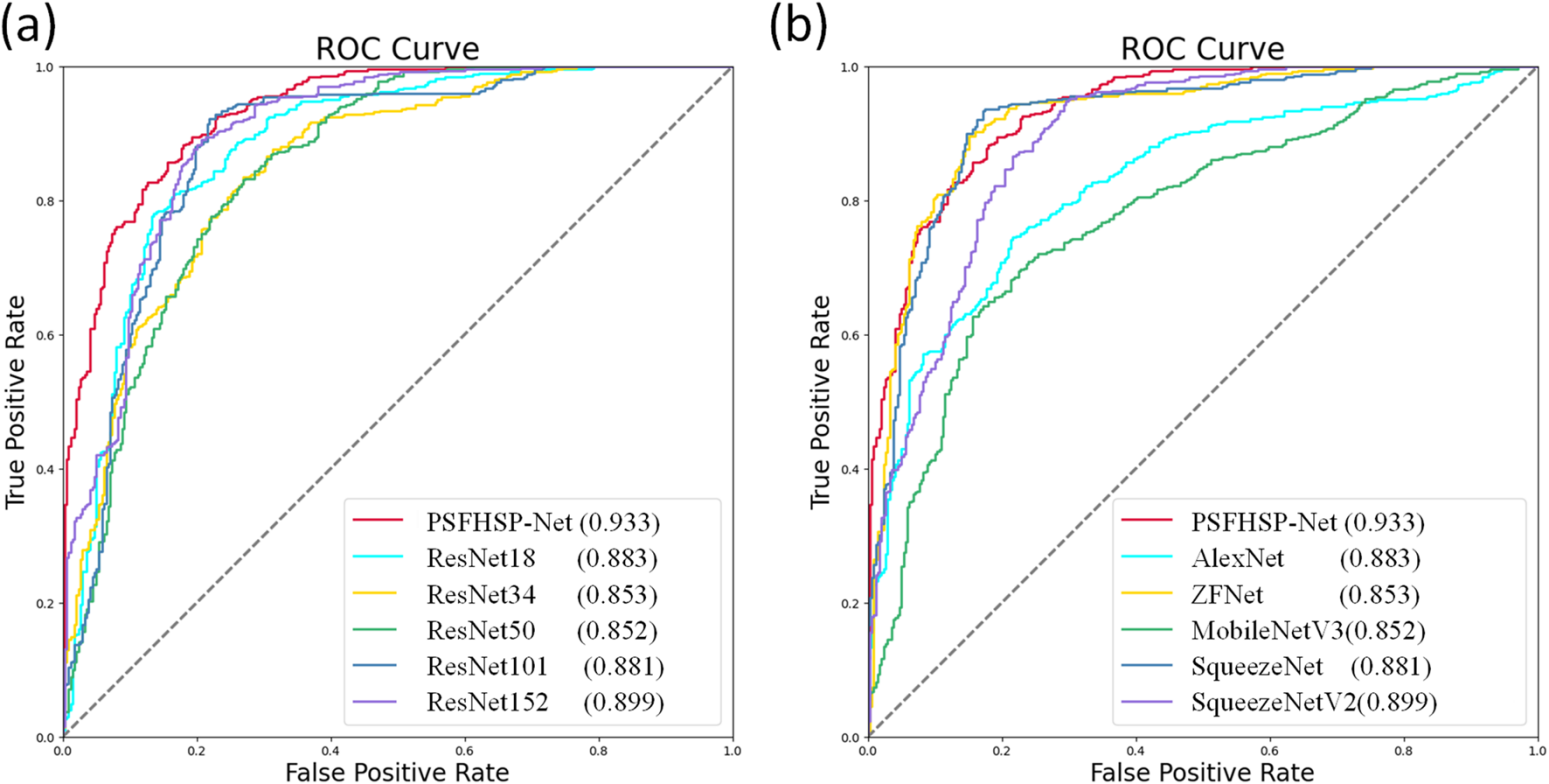
ROC curves of different methods. **a** AUC of PSFHSP-Net is compared with those of non-lightweight models. **b** AUC of PSFHSP-Net is compared with those of lightweight models. ROC, receiver operating characteristic

For a more quantitative comparison, ROC curves for all models, both non-lightweight and lightweight networks, regarding the PSFHSP recognition task are depicted in Fig. 4. Relative to non-lightweight models (Fig. 4a), the AUC value of PSFHSP-Net (0.933) is superior to those of ResNet-18, ResNet-50, ResNet-101, and ResNet-152. When compared with lightweight models (Fig. 4b), PSFHSP-Net’s AUC also exceeds those of AlexNet, ZFNet, MobileNetV3, SqueezeNet, and ShuffleNetV2. An AUC of 0.933 and a corresponding recall of 0.9181 indicate that PSFHSP-Net can effectively identify most standard planes, demonstrating excellent generalization capability to meet clinical requirements and showcasing its potential applicability and clinical value.

**Fig. 5:**
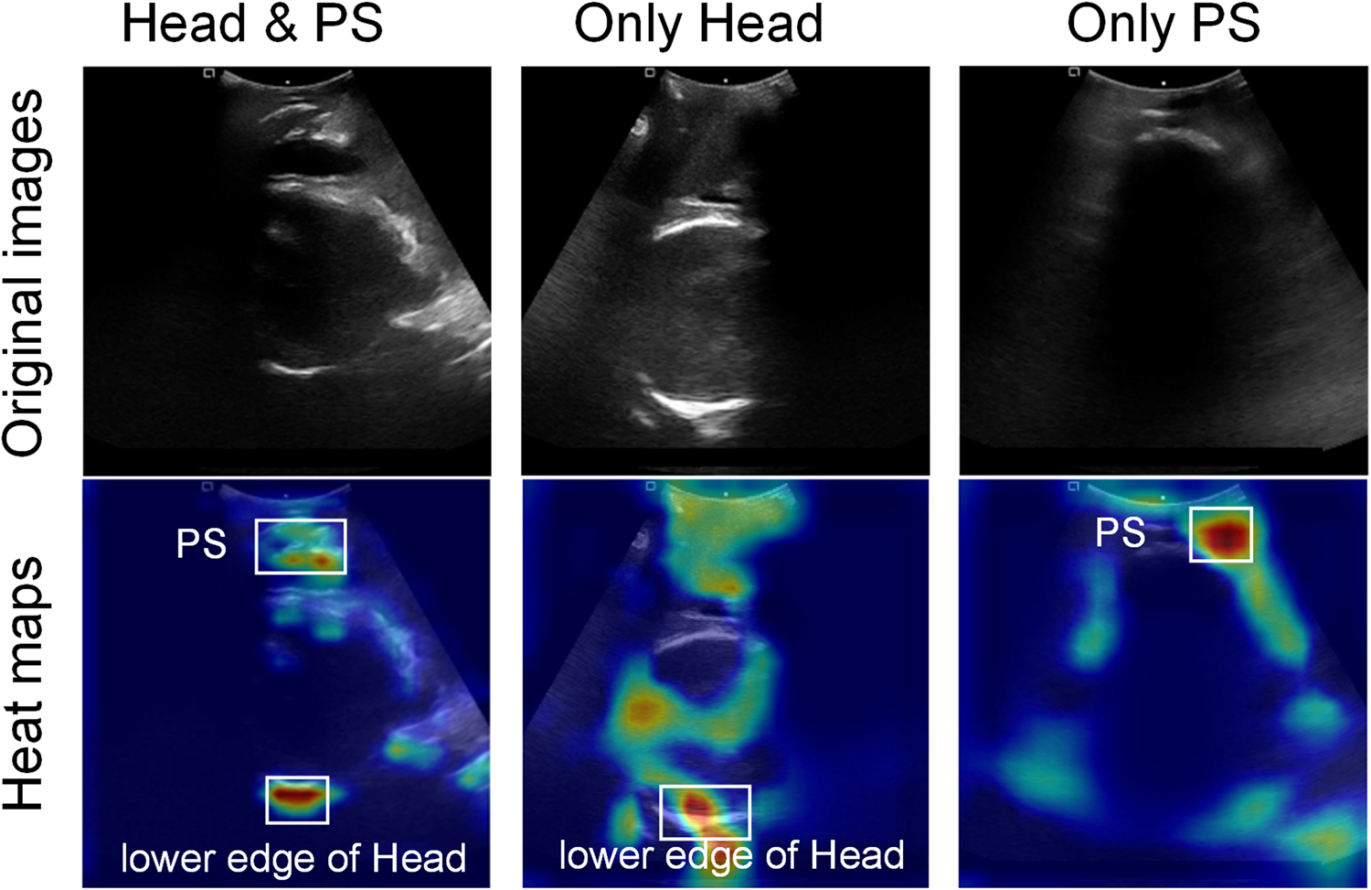
Examples of correctly classified images and their heatmaps. For PSFHSP: Head & PS, the model’s attention is focused on the PS structure and the lower edge of the fetal head. For Non-PSFHSP: Only Head and Only PS, the model could detect the lower edge of the fetal head and the pubic symphysis, respectively

**Fig. 6:**
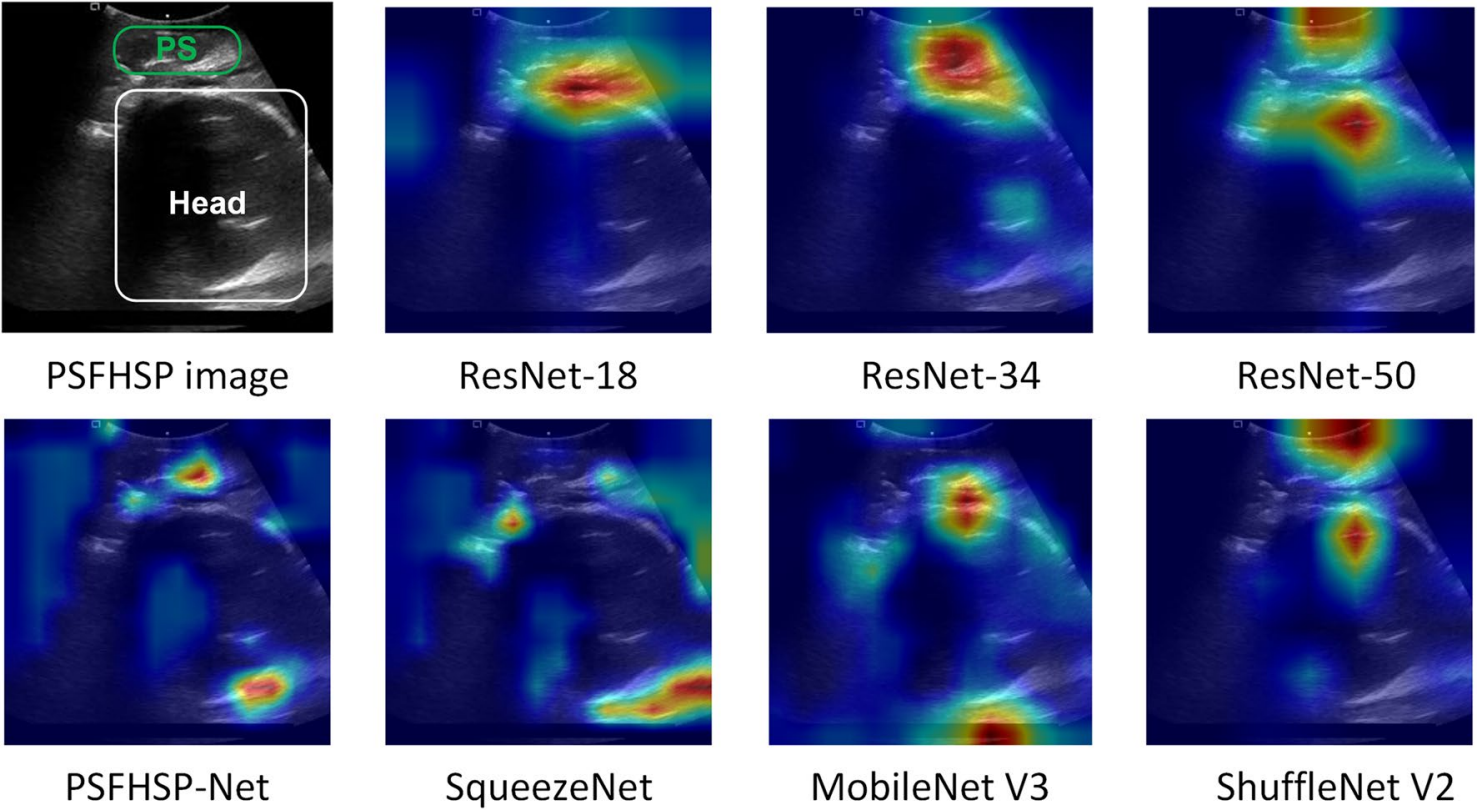
Examples of PSFHSP image and predictive heat maps of some models

To verify the effectiveness of PSFHSP-Net, we employed the Grad-CAM algorithm to visualize the weights of feature maps in the form of heatmaps. Based on the red areas in the heatmaps, we analyzed whether the network had accurately learned the critical features or information. Figure 5 displays the PSFHSP image and its corresponding heatmap. The heatmap for PS & Head shows that the model’s focus is appropriately on the pubic symphysis structure and the lower edge of the fetal head. The heatmap for Only Head reveals that the model does not detect the pubic symphysis, concentrating instead on the lower edge of the fetal head. Conversely, in the heatmap for Only PS, the model detects the fetal head but not the pubic symphysis. These results are consistent with their respective ground truths, confirming that the model effectively learns essential structures. Figure 6 illustrates the PSFHSP image and thermal maps for models with performance similar to PSFHSP-Net, showing that PSFHSP-Net accurately detects both the fetal head and the pubic symphysis, unlike other models that detect only one of these structures.

To further demonstrate the effectiveness of our modifications, we conducted ablation experiments. ResNet-18 comprises a four-tier structure, while PSFHSP-Net utilizes the first tier of ResNet, as well as the first block in the second layer. Our experiments explored structures containing three layers, two complete layers, and a single layer. A downsampling module was added at the end of the second layer in PSFHSP-Net, and ablation studies on this modification were also performed. As listed in Table 3, PSFHSP-Net achieved ACC, *F*1-score, and recall of 0.8995, 0.9075, and 0.9181, respectively, with variances in these metrics during five-fold cross-validation lower than in other configurations. The average recall rate was 0.9022 with a variance of 0.0857, slightly lower than that of the single-layer model (mean of 0.9142 and variance of 0.0185) but not significantly so. Despite the single-layer model’s smaller size, PSFHSP-Net demonstrated superior performance in all other metrics and comparable frame rates. The inclusion of a downsampling module significantly improved all aspects of PSFHSP-Net compared to PSFHSP-Net without the downsampling module. Comprehensive comparisons confirm that PSFHSP-Net is the most suitable compression method for PSFHSP classification within the ResNet-18 framework.

**Table 3:** The performance of different compressions for ResNet18 on the test dataset. The best results are in bold

Model	ACC	*F*1-score	Recall	Precision	Parameters (MB)	Frames per second (FPS)
With_onelayer	0.8371 ± 0.0485	0.8701 ± 0.0393	0.9171 ± 0.0880	0.8313 ± 0.0471	0.60	**67.8785 ± 3.9073**
With_twolayers	0.8217 ± 0.0440	0.8431 ± 0.0460	0.8179 ± 0.0943	0.8773 ± 0.0256	2.61	64.7592 ± 6.7393
With_threelayers	0.7998 ± 0.0482	0.8109 ± 0.0570	0.7355 ± 0.0976	**0.9142 ± 0.0185**	10.62	63.3086 ± 3.0739
PSFHSP-Net_w/oDS	0.8551 ± 0.0426	0.8538 ± 0.0351	0.8264 ± 0.0872	0.8425 ± 0.0416	**1.48**	65.8664 ± 2.2310
PSFHSP-Net	**0.8995 ± 0.0426**	**0.9075 ± 0.0282**	**0.9181 ± 0.0679**	0.9022 ± 0.0857	1.51	65.7909 ± 1.9000

With_onelayer, the model consisting of the first layer of ResNet18; With_twolayers, the model consisting of the first two layers of ResNet-18; With_threelayers, the model consisting of the first three layers of ResNet18; and PSFHSP-Net_w/oDS, the model is PSFHSP-Net without the downsampling module

## Discussion

In this study, we introduce an innovative automatic PSFHSP recognition method that simplifies model depth and modifies residual modules to enhance classification precision. The proposed PSFHSP-Net is a lightweight, end-to-end solution designed to facilitate the quick and effective screening of the PSFHSP, thereby supporting accurate AoP measurements. With fewer parameters and rapid inference speeds, our model enables sonographers to efficiently identify PSFHSP, providing a reliable foundation for subsequent AoP evaluation.

PSFHSP recognition is technically straightforward in terms of model complexity. Deep learning techniques are proficient at detecting key structures such as the pubic symphysis and fetal head in ultrasound images, determining the presence of PSFHSP [25]. In these images, features like the fetal head and the marginal structure of the pubic symphysis are relatively superficial and easily discernible to the human eye [26]. Consequently, these features can be extracted with just a few convolutional layers. In contrast, models with excessive depth, such as ResNet101 or ResNet152, may not only capture these superficial features but also learn to recognize insignificant, irrelevant, or even detrimental features. Given the relationship between these extraneous features and noise artifacts in ultrasound images, increasing model depth does not necessarily correlate with enhanced performance [27]; indeed, as models deepen from ResNet-18 to ResNet-101, classification accuracy has been observed to decline from 0.8560 to 0.7571, as shown in Table 2. Notably, our proposed PSFHSP-Net, with only three layers, achieved the highest accuracy of 0.8995.

**Fig. 7:**
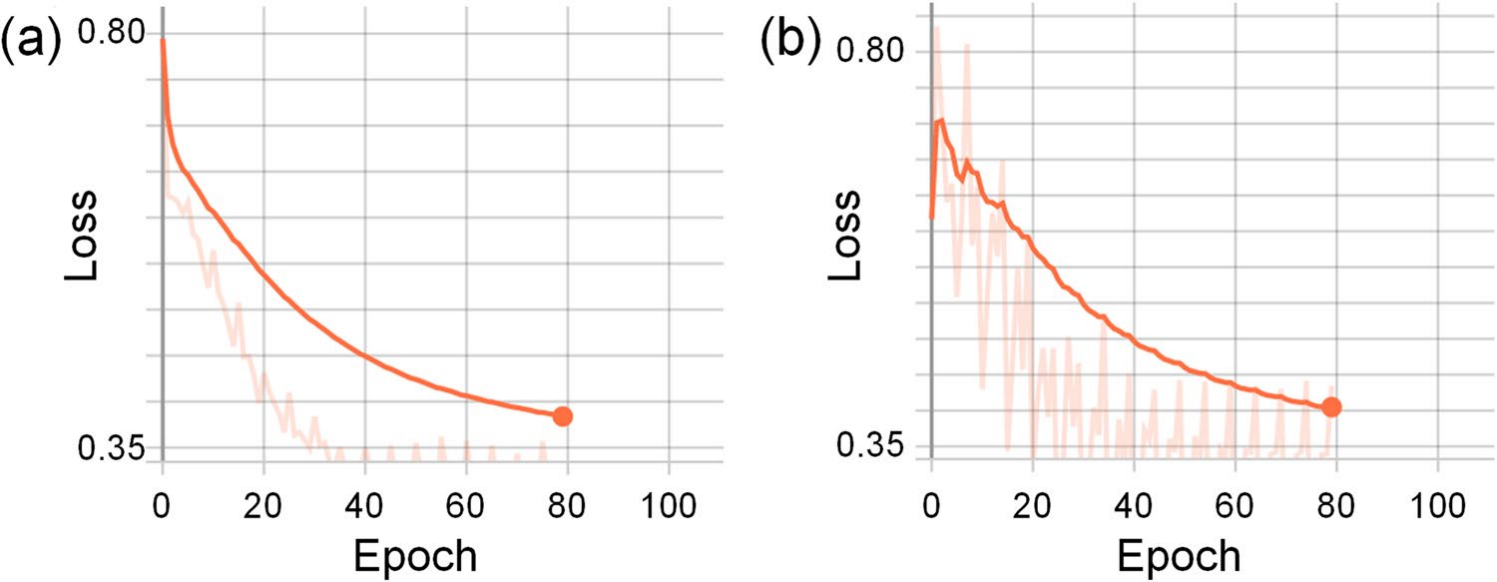
Training loss **(a)** and validation loss **(b)** of the network versus the number of epochs. There is no overfitting, because the early stop strategy was used during the training

The effectiveness of PSFHSP-Net can be attributed to the inclusion of residual blocks, which prevent network degradation—a common issue in non-residual models like AlexNet and ZFNet, where performance typically stagnates (Fig. 7a, b). In contrast, the residual model ResNet-18, with just 18 layers, saw a classification accuracy improvement of over 19% compared to non-residual models. PSFHSP-Net utilizes three residual blocks, a pooling layer, and a fully connected layer. Compared to ResNet-18, PSFHSP-Net has significantly fewer parameters, approximately 2800% less, which contributes to a 4% increase in classification accuracy and a 7% increase in inference speed.

PSFHSP-Net’s streamlined architecture not only reduces parameter count but also enhances inference speed, allowing it to process over 65 images per second and meet real-time requirements. This makes it suitable for deployment on mobile ultrasound devices. The automatic nature of the model simplifies the clinical workflow by directly processing scanned video into images that are immediately analyzed for PSFHSP, eliminating the manual screening process and improving productivity in AoP measurements.

**Fig. 8:**
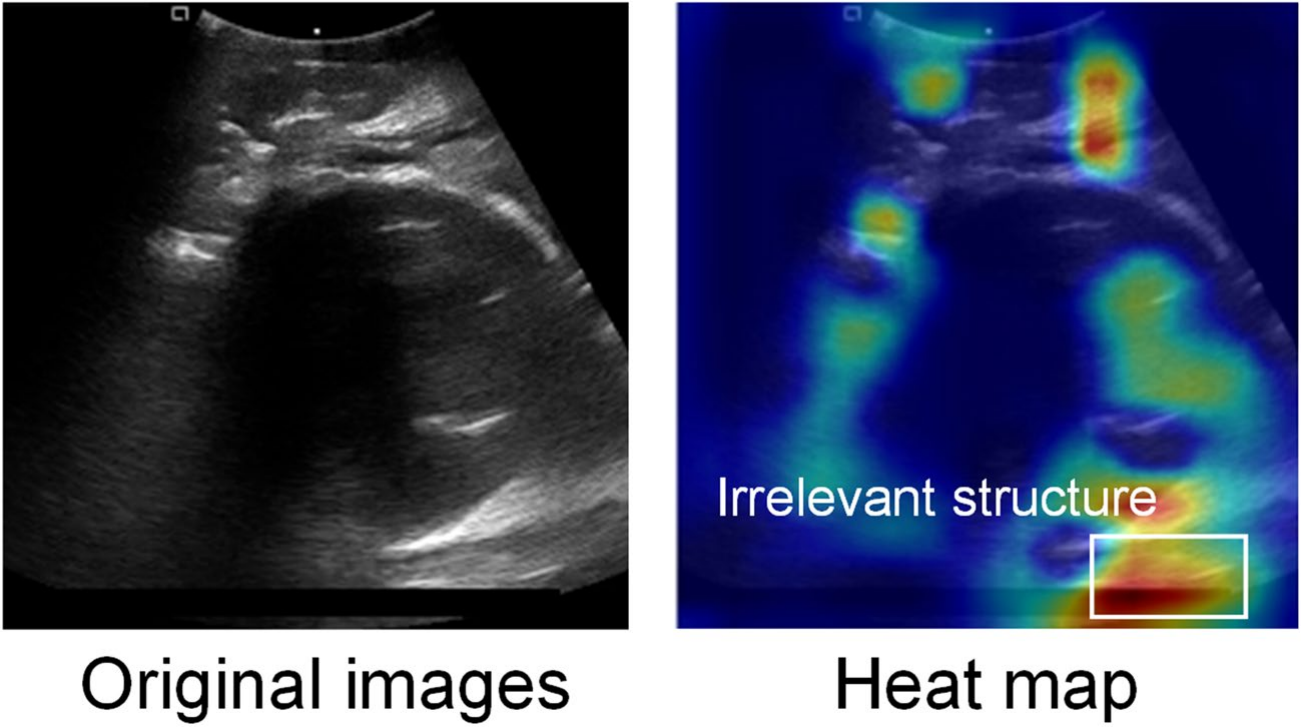
The impact of shadows and noise on the performance of PSFHSP-Net. Due to the influence of the inherent mechanism of imaging in medical US images, there are many speckles, noise, and artifacts, which affect the image quality. Therefore, in the training process, the model was prone to mistake some shadows and noise as key results for learning

Despite its strengths, PSFHSP-Net can still be improved. Ultrasound images often contain speckles, noise, and artifacts that degrade image quality and can mislead the learning process [28]. For instance, tests have shown that parts of the model’s weights may focus erroneously on artifact areas, misidentifying irrelevant bright spots as key structural edges (Fig. 8). To address these challenges, future developments will include integrating a dedicated denoising module into the model's architecture to cleanse the images before feature extraction and classification. This enhancement is expected to refine the model’s performance and robustness against noise interference [29, 30]. Additionally, we plan to implement an attention mechanism that prioritizes critical information (i.e., fetal head and pubic symphysis) while disregarding irrelevant details (such as noise and shadows) [31]. This approach will adaptively adjust weights to continuously select essential data under varying conditions, thereby improving the model’s robustness and accuracy and reducing the impact of noise and artifacts on classification performance [32, 33].

## Conclusions

In summary, this study presents an innovative approach to automatically recognizing the PSFHSP in ultrasound imaging. By compressing the depth and modifying the residual modules of the model, we have enhanced its classification precision. Unlike traditional deep learning models, which typically require significant computational resources and substantial energy consumption, our proposed model is optimized for real-time classification on resource-constrained and low-energy edge devices. This makes it particularly well-suited for use with portable, handheld intrapartum ultrasound machines, offering a significant advancement in the field by facilitating efficient and reliable diagnostic assessments in various clinical environments.

## Abbreviations

US: Ultrasound
AoP: Angle of progression
CNN: Convolutional neural network
PSFHSP: Pubic symphysis-fetal head standard plane
PS: Pubic symphysis
PSFHSP-Net: Pubic symphysis-fetal head standard plane-network
BN: Batch normalization
ACC: Accuracy
ROC: Receiver operating characteristic
AUC: The area under the receiver operating characteristic curve
